# Exploring resource availability for nurses implementing HIV prevention guidelines in primary healthcare facilities

**DOI:** 10.4102/hsag.v30i0.3084

**Published:** 2025-08-21

**Authors:** Junior M. Ntimani, Andile G. Mokoena-de Beer, Deliwe R. Phetlhu

**Affiliations:** 1 Nursing Sciences Department, School of Health Sciences, Sefako Makgatho Health Sciences University, Pretoria, South Africa

**Keywords:** PrEP, guidelines, implementation, nurses, resources, support

## Abstract

**Background:**

Pre-exposure prophylaxis (PrEP) lowers new human immunodeficiency virus (HIV) infections among individuals at risk; however, its uptake in South Africa is hindered by resource limitations within public health facilities. This occurs despite the established PrEP guidelines to promote its use.

**Aim:**

This study aimed to explore the availability of resources that support nurses in implementing the PrEP guidelines in Johannesburg’s primary health settings.

**Setting:**

Four primary healthcare settings in sub-districts A and E of the City of Johannesburg, South Africa, were used to conduct the study.

**Methods:**

A qualitative exploratory design with an interpretive approach was used to gather insights into the availability of resources for implementation of PrEP guidelines. Donabedian’s framework was used to assess implementing PrEP guidelines looking at the structure, process and outcomes linked to resource availability. Data were gathered from 19 nurses in four primary healthcare facilities via semi-structured interviews and analysed using the thematic analysis method.

**Results:**

Three overarching themes emerged as barriers to effective PrEP implementation: (1) structural inadequacies; (2) healthcare system processes and support; and (3) unclear performance tracking. Both barriers and facilitators were identified to have an impact on the implementation of PrEP while highlighting the need for the strengthening of the healthcare system in HIV prevention success.

**Conclusion:**

The study highlights critical resource limitations hindering PrEP implementation. It underlines the urgent need for improved physical infrastructure, additional human resources and robust data management systems.

**Contribution:**

The study emphasises the need for policymakers to strengthen infrastructure and human resources to minimise service delays and inefficiencies, ultimately reducing healthcare costs by enhancing PrEP uptake and retention.

## Introduction

Human immunodeficiency virus (HIV) prevention is a critical pillar in South Africa’s efforts to manage the HIV epidemic and ensure HIV-free generation by 2030 (Faro et al. 2022). The current HIV testing and treatment targets are 95-95-95 strategy, and they must be met by 2025 to eliminate AIDS by 2030 (UNAIDS 2021). The 95-95-95 strategy is a set of ambitious targets released by UNAIDS in 2020 (Frescura et al. 2022). This strategy calls for 95% of all people living with HIV to know their status, 95% of people diagnosed with HIV to be on treatment and 95% of all people receiving Antiretroviral Therapy (ARVs) to be virally suppressed by 2025 (UNAIDS 2021). In 2024, South Africa indicated that all individuals living with HIV were aware of their HIV-positive status. Among this population, 79% were undergoing antiretroviral treatment (ART), with 93% of those on ART achieving viral suppression (SANAC 2024). This indicates significant advancements in HIV testing and treatment, yet it also underscores persistent deficiencies in the HIV care cascade. Of those aware of their status, 21% are not receiving antiretroviral therapy (ART), while an additional 7% of individuals on ART are not achieving viral suppression. This suggests that a considerable percentage of individuals living with HIV, who are aware of their status, exhibit unsuppressed viral loads and may persist in transmitting the virus, especially without the implementation of pre-exposure prophylaxis (PrEP) and regular condom usage (Cohen et al. 2016; UNAIDS 2022). These gaps may arise from issues including delays in treatment initiation, inadequate adherence or the pressures faced by overburdened and understaffed healthcare facilities.

Pre-exposure prophylaxis consists of the antiretroviral medication emtricitabine (FTC) and tenofovir (TDF), which are administered to HIV-negative individuals at risk of HIV acquisition. It is commenced before and maintained throughout times of potential HIV exposure to optimise its preventive efficacy (WHO 2023). The PrEP guideline was integrated into the South African health system in 2016, in accordance with the World Health Organization’s recommendation for its implementation as an HIV prevention strategy. It was initially provided to specific groups of individuals such as men sleeping with men (MSM), prisoners and female sex workers (FSWs). However, in 2017, PrEP services were expanded to a greater population, including serodiscordant couples and people who perceive themselves to be at risk of acquiring HIV (National Department of Health [NDoH] 2019). Presently, this HIV prophylaxis is provided as a component of a comprehensive package of services that include voluntary medical male circumcision, HIV testing, risk reduction counselling, condoms for both sexes, lubricants and ARV treatment for partners who are HIV positive (NDoH 2021). Pre-exposure prophylaxis is 99% effective against HIV during sexual intercourse and 75% effective on people sharing drug injection equipment (Janes et al. 2018). Because of its efficiency, PrEP has the potential to significantly contribute to South Africa’s goal of achieving an HIV-free generation by 2030. However, this prophylaxis is underutilised (Faro et al. 2022).

The Tembisa model, which analyses South Africa’s HIV epidemic, indicates an upward trend in PrEP utilisation among sexually active adults. In 2018, the usage of PrEP was at 0.1%, which increased to an estimated 0.8% by 2022 (Johnson et al. 2022). Furthermore, sexually active adolescent girls and young women made up 4% of those taking PrEP in 2022, up from 0.1% in 2018. While these increases are undoubtedly a step in the right direction, and the clear benefits of PrEP for everyone who has access to it are obvious, uptake is not adequate. Uptake needs to be substantially higher for it to start having a significant effect on the country’s infection rates (Bekker, Gill & Beyrer 2022).

In South Africa, PrEP is provided at no cost in all primary healthcare (PHC) facilities. However, its uptake is still low in relation to the rate of new HIV infections (NDoH 2023; Venter et al. 2022). Resources encompass the assets, personnel, materials and systems utilised to attain healthcare objectives (Armstrong & Taylor 2014). The Tembisa model directs the examination of obstacles to PrEP implementation. Challenges to effective PrEP implementation are complex and can be categorised into system-level, provider-level and patient-level barriers. Furthermore, the incorporation of PrEP into the comprehensive HIV treatment framework, especially within ART programmes, necessitates strategic planning, sufficient infrastructure, increased human resources and well-defined guidelines (Mugwanya et al. 2021). Numerous healthcare facilities are constrained by insufficient physical space to provide PrEP services confidentially, thereby restricting their accessibility and acceptability (Frescura et al. 2022; SANAC 2024). Furthermore, the lack of clarity regarding the specific allocation of resources for PrEP complicates service delivery (Chimbindi et al. 2022).

Nurses responsible for implementing PrEP often report significant workloads within already strained health systems (Pleaner et al. 2023). In the absence of dedicated PrEP personnel, providers are required to administer PrEP in conjunction with other clinical responsibilities, including ART, antenatal care and general outpatient services. The workload diminishes the time allocated for PrEP-specific counselling and follow-up, impacting provider motivation and the overall quality of care (Mayer, Agwu & Malebranche 2020). Despite the introduction of strategies such as task shifting and task sharing to alleviate this burden, their implementation continues to be inconsistent (Court et al. 2024).

Patient-level uptake is impeded by low self-perceived HIV risk, stigma and inadequate health literacy, particularly in under-resourced urban communities (Schwartz et al. 2019; Velloza et al. 2020). Despite heightened awareness of PrEP, misconceptions and fear of judgement persist, hindering individuals from commencing PrEP use. The combination of patient-related barriers and systemic and provider-level constraints leads to the continued underutilisation of this effective HIV prevention tool. Gaps in staff training, infrastructural inadequacies and misalignment of resources underscore the necessity for targeted interventions to support nurses and improve PrEP uptake at the facility level (Mukwena & Manyisa 2022).

## Problem statement

In 2016, South Africa adopted the WHO recommendations to utilise PrEP as an HIV prevention strategy. Despite this initiative, the City of Johannesburg (CoJ) reported a rise in HIV-positive cases, with 756 756 individuals testing positive in 2020, up from 715 391 in 2017 (City of Johannesburg 2020). Additionally, approximately 685 people aged 15 to 49 become infected with HIV daily in South Africa (Allinder 2020). Since the implementation of these guidelines, PrEP uptake has been suboptimal because of limited awareness and education, stigma and discrimination and adherence challenges. The successful implementation of PrEP guidelines necessitates a consistent supply of PrEP medications, the availability of skilled staff including physicians and nurses, adequate healthcare infrastructure and effective supporting services such as laboratory facilities. However, challenges persist in the availability of these essential resources despite the assumption that Metropolitan cities are highly resourced. Considering these challenges, the study sought to explore the availability of resources required to support nurses in the implementation of PrEP guidelines in Johannesburg’s PHC facilities.

### Aim of the study

The study explored the availability of resources required to support nurses’ implementation of PrEP guidelines in Johannesburg’s PHC facilities.

### Research methods and design

The researchers used a qualitative exploratory design that is interpretive in nature, which helped to gather new insights that could be valuable to shape the implementation of PrEP guidelines in PHC settings (Flanagan & Beck 2025). The study was grounded by Donabedian’s framework of assessing healthcare quality to explore resource availability for nurses implementing PrEP guidelines. This framework examines healthcare delivery by observing the functionality and effectiveness of the health facilities’ structure, process and related outcomes (Donabedian 1980).

### Setting of the study

This study was conducted in municipal clinics located within the CoJ’s sub-districts A and E. Each sub-district comprises eight fixed PHC facilities, resulting in a total of 16 clinics. Of these, four clinics were purposively selected as data collection sites based on their high burden of HIV-positive patients. These clinics are fixed-site PHC clinics that operate weekdays from 07:00 to 16:00, with extended hours on Saturdays from 07:00 to 13:00 to accommodate patient demand. The strategic plan document of CoJ (2021) noted an estimated population of 5.87 million, of which 783 032 were people living with HIV by 2019.

The CoJ, one of the largest metropolitan municipalities in South Africa, is not only predominantly urban but also encompasses peri-urban and semi-rural zones within its municipal boundaries. The selected clinics receive external technical and programme implementation support from the Anova Health Institute, a non-governmental organisation funded by international donors to strengthen HIV and PrEP service delivery in public health settings.

### Population and sampling

The population comprised registered nurses working in the four selected clinics. These registered nurses were trained in Nurse-Initiated Management of Antiretroviral Therapy (NIMART) and the PrEP guidelines. A purposive sampling technique was employed to recruit registered nurses for the study. The participants had to be registered nurses, with experience of working at a facility providing ART or PrEP services for at least 6 months to be included in the study. Nurses who were newly appointed (less than 6 months in the role), those not directly involved in ART or PrEP service delivery and administrative staff were excluded from the study. Initially, 34 registered nurses were recruited for the study; however, data saturation was achieved at 18 and confirmed at the 19th participant.

### Participants’ recruitment

Participants were recruited voluntarily with the assistance of facility operational manager, free from any coercion. They were apprised of the study’s objective, their roles and their rights, including the option to withdraw from the study at any point without repercussions. Ethical guidelines were rigorously followed, ensuring that all participants gave informed consent prior to participation.

### Data collection instrument development

A semi-structured interview guide was specifically designed to correspond with the Donabedian framework of healthcare quality. This framework evaluates healthcare delivery with particular focus on the dimensions of structure, process and outcome, serving as a foundational framework for the design and organisation of the interview questions. The guide included predefined, open-ended questions that enabled participants to elaborate on specific topics, while also allowing for probing and clarification. Furthermore, the interview guide included a demographical data section, which was used to provide a brief description of the participants.

The guide was developed through a systematic multi-step process. A thorough literature review was performed to identify significant thematic areas and resource-related challenges in the implementation of PrEP. Subsequently, feedback was obtained from registered nurses to confirm that the questions were pertinent, contextually appropriate and practically applicable. Each section of the guide aligned with one of the three Donabedian components: structure-related questions examined physical infrastructure, human resources and access to mentors; process-related enquiries investigated data management practices, training on PrEP and ART guidelines, availability of clinical protocols and referral pathways; outcome-focused questions assessed perceived trends in PrEP uptake and retention within the facilities. Three pilot interviews were conducted in two selected clinics to test the feasibility of the developed interview guide. The pilot interviews confirmed that the guide effectively captured relevant data across all three domains of the Donabedian model. As such, no amendments were made, and the pre-test data were included in the main analysis as they aligned well with the study objectives.

### Data collection

Semi-structured individual face-to-face interviews were conducted between March and May 2024. Each interview lasted between 30 min and 45 min. The researcher visited the selected clinic on the scheduled days for data collection. Participants were asked three central questions: ‘*What is the outcome of the current PrEP guideline implementation in your facility*?’, ‘*What challenges have you encountered during the implementation of PrEP guidelines*?’ and ‘*How is the implementation of PrEP guidelines progressing in your facility*?’ Additional probing questions were used to gather deeper insights. Interviews were conducted in English and held in a private office. Descriptive field notes were taken, and with the participants’ permission, an audiotape was used to record the conversations. The interviews were finalised with the 19th participant, as data saturation was reached at the 18th participant and validated by the 19th, with no additional information arising.

### Data analysis

Data analysis is a systematic process of examining and interpreting data to uncover patterns, relationships and insights (Creswell 2018). Prior to analysis, the data were transcribed verbatim from the audio recordings. The transcribing procedure guaranteed that every aspect of the interviews was precisely documented, allowing for a complete thematic analysis by the researcher and the independent coder (Riger & Sigurvinsdottir 2016). Thematic analysis allowed the researchers to familiarise themselves with the data, code it, identify themes and sub-themes, interpret the results and report the findings. The collected data consisted of text from descriptive field notes taken during the interviews and audio recordings. An experienced qualitative data analysis specialist, together with the researcher, confirmed the emerging themes and sub-themes through a consensus meeting.

### Measures to ensure trustworthiness

The aim of trustworthiness in qualitative inquiry is to support the argument that the findings are worth paying attention to (Patton 2015). To ensure the truthfulness, appropriateness and meaningfulness of the data, measures were taken according to the four criteria of credibility, transferability, conformability and authenticity (Polit & Beck 2017). Credibility was achieved by triangulating methods of data collection using interviews, fieldnotes and observations. Member checking was also applied, where the transcribed data were taken back to the participants to ensure it kept the meaning as shared by them (Flanagan & Beck 2025). Transferability was by providing a dense description of the setting where data were gathered including description of the participants as well as the methods applied to this study. Furthermore, confirmability was achieved by grounding the research findings in objective evidence, which directly reflects the resources required for nurses to implement PrEP guidelines. This was accomplished via thorough data analysis and triangulation, comparing participant responses, field notes and external data sources to ensure consistency and alignment with the study’s objectives. The alignment of findings with practical guidelines and tools substantiated their applicability. The inclusion of an independent coder in the thematic analysis process was a crucial strategy for ensuring confirmability.

### Ethical consideration

Ethical approval to conduct the study was obtained from the Sefako Makgatho Health Sciences University Research Ethics Committee (reference no: SMUREC/H/485/2023: PG), the Johannesburg Health District Research Ethics Committee (NHRD REF.NO.:GP_202402_012) and the PHC managers where the study took place. The participants were approached directly to explain the purpose of the study. Thereafter, individual appointments were made with those who demonstrated interest to obtain written informed consent. During data gathering process, the identity of participants was withheld, and data were protected using password encryption ensuring privacy and anonymity. Furthermore, participants were assigned codes to ensure no data are linked to them, thus ensuring confidentiality. Their right to self-determination was maintained as they were informed of their right to withdraw at any time without consequences at the beginning of the study. No participant experienced harm when gathering data; however, the researchers were ready to provide debriefing sessions in the event where participants would experience emotional discomfort.

## Results

### Description of demographics

Participants’ ages ranged between 24 years and 64 years, with a mean age of 35.3 years for males and 40.6 years for females. A total of 19 registered nurses participated in the study. Of the 19, 3 (15.7%) were males and 16 (84.3%) were females. All participants (100%) were NIMART trained, while 17 (89.5%) of them were trained for PrEP, see Table 1 below.

**TABLE 1:** Characteristics of the participants.

Participant no	Gender	Age (years)	Experience in years	PrEP training	NIMART training
Participant 1	Female	37	10	No	Yes
Participant 2	Female	27	3	Yes	Yes
Participant 3	Female	49	7	Yes	Yes
Participant 4	Female	36	6	Yes	Yes
Participant 5	Female	64	10	Yes	Yes
Participant 6	Female	60	14	Yes	Yes
Participant 7	Female	45	8	Yes	Yes
Participant 8	Male	31	5	Yes	Yes
Participant 9	Male	32	6	Yes	Yes
Participant 10	Female	31	4	Yes	Yes
Participant 11	Female	24	2	Yes	Yes
Participant 12	Female	31	4	Yes	Yes
Participant 13	Female	31	3	Yes	Yes
Participant 14	Female	52	6	Yes	Yes
Participant 15	Male	43	5	Yes	Yes
Participant 16	Female	48	7	Yes	Yes
Participant 17	Female	28	5	Yes	Yes
Participant 18	Female	31	4	Yes	Yes
Participant 19	Female	49	13	No	Yes

PrEP, pre-exposure prophylaxis; NIMART, nurse-initiated management of antiretroviral therapy.

### An overview of the themes

Guided by Donabedian’s framework, three overarching themes emerged as barriers to PrEP implementation: (1) structural inadequacies, (2) healthcare system processes and support and (3) unclear performance tracking, see Table 2.

**TABLE 2:** Themes and sub-themes.

Donabedian’s Concept measure	Themes	Sub-themes
Structure	1. Structural inadequacies	1.1 Inadequate physical infrastructure 1.2 Shortage of human resources 1.3 Availability and accessibility of clinical mentors and guidelines
Process	2. Healthcare system processes and support	2.1 Poor data management 2.2 Availability of clinical guidelines 2.3 The role of training on PrEP or ART guidelines 2.4 Access to effective referral system and laboratory services
Outcomes	3. Unclear performance tracking	3.1 Uncertainty regarding facility PrEP targets

PrEP, pre-exposure prophylaxis.

#### Theme 1: Structural inadequacies (structure)

Participants indicated that there are structural challenges that hinder the implementation of PrEP guidelines in PHC settings. These are reflected as inadequate physical infrastructure and a shortage of human resources. However, the availability and accessibility of clinical mentors and guidelines assisted them to mitigate the challenges they experienced while implementing PrEP guidelines. These focused on how the structural aspects of the facilities impact the implementation of the PrEP guideline and, consequently, patient care.

**Inadequate physical infrastructure:** Participants reported that the clinics were small and reflected on the impact of the facility’s physical structure. These included the number of consultations rooms and the structural condition of some facilities. They reported that inadequate infrastructure might adversely affect the implementation of PrEP guidelines by restricting accessibility, impairing workflow efficiency and possibly reducing patient trust and acceptance of PrEP service. The following are verbal quotations in support of these findings:

‘The clinic is quite small compared to the numbers of patients that we are seeing including those on PrEP. The consulting rooms are not enough as well.’ (P2, 27-year-old, Female)

‘There’s a limited capacity and space. Our clinic is very small. HTS counsellors work in a very dilapidated small container, and it cannot carry a lot of patients so those are our concerns.’ (P11, 24-year-old, Female)

‘Our clinic is very old and small. It cannot accommodate more patients because already this space is not enough for existing patients.’ (P2, 27-year-old, Female)

**Shortage of human resources:** Participants indicated that the primary challenge in implementing PrEP guidelines was a shortage of staff. They emphasised that inadequate staffing significantly hindered the effective rollout of PrEP, resulting in poor PrEP uptake and retention, and an increased workload. The following quotes were provided to support these claims:

‘Shortage of staff, shortage stationery, shortage of medication, and poor compliance with treatment from patients.’ (P3, 49-year-old, Female)

‘We don’t have enough human resources, especially when it comes to clinicians [*professional nurses*] because our Total Number of Patients Remaining on ART [*TROA*] is around 3500 to 3800 and we have got about 6 clinicians who are working in this facility. So, you can imagine the number of patients who are coming in. We are not talking about Comprehensive Care, Management and Treatment [*CCMT*] patients only, we have other services to render like curative, ANC, mother and child, and the emergencies.’ (P8, 31-year-old, Male)

‘Staff shortage, especially with professional nurses. It becomes difficult to cope with workload of patients especially when other nurses are on leaves.’ (P1, 37-year-old, Female)

**Availability and accessibility to clinical mentors:** Participants highlighted the substantial impact of clinical mentors on their capacity to manage patients effectively. In this study, clinical mentors refer to experienced professional nurses or clinical advisors assigned by the Department of Health and implementing partners to support frontline healthcare workers in HIV and PrEP service delivery. The presence of clinical mentors provided essential guidance and support, leading to enhanced patient care. The mentors were present in the facilities on designated days, offering practical support and directly addressing clinical challenges. During instances of physical absence, participants had the option to contact their mentors through telephone, thereby maintaining ongoing support and consultation as required. Participants had the following to say:

‘They [*Clinical mentors*] explained to us that they are unable to come to the facility monthly, so it’s best if they come at least once a quarter and if there are any challenges, we reach out to them telephonically and they are quick to assist us.’ (P7, 45-year-old, Female)

‘We do have mentors. However, they visit the clinic on certain day. When they are not in the clinic and we have challenges with patients, we call them and have a telephonic consultation.’ (P8, 31-year-old, Male)

#### Theme 2: Healthcare system processes and support (process)

Participants indicated that there is a poor data management process that hinders the implementation of PrEP guidelines; however, they also highlighted that sufficient resources and support play a significant role in successfully implementing PrEP in accordance with the established guidelines for its rollout to a broader population.

**Poor data management:** Poor data management was identified as a barrier to PrEP implementation in PHC facilities. Participants in this study remarked on the system utilised for capturing of PrEP patients and reporting to the next level. Participants reported that the system is flawed, providing wrong data, thus affecting the performance of the clinic. This is reflected in the following quotes:

‘I don’t think all patients are appearing in the system. At times, you may initiate a patient, but when you try to retrieve their file 28 days later, the file is missing, and you have no way of knowing where it is. In such cases, I would ask the data capturing team if they had recorded this patient, only to find that the patient does not appear in the system.’ (P14, 42-year-old, Female)

‘Filling is haphazard. When a patient comes back for review, you do not find the original file. Its either you use a continuation sheet or make a duplicate file; you don’t have the patient history and most of patients are not captured on tier.net system.’ (P11, 24-year-old, Female)

**Availability of clinical guidelines:** Participants cited that having the PrEP guidelines readily available in the facility served as a facilitator for implementing PrEP guidelines. This helps the PHC facilities to follow the steps for implementing PrEP correctly. It simplifies the process and ensures that they provide care the way it is intended. Participants had the following to say:

‘Each room is expected to have a copy of the guidelines, but we are also permitted to use soft copies. Since hard copies are not always readily available, we have a clinic WhatsApp group where these guidelines are shared, making them easy to access. This includes the latest ART guidelines as well as the PrEP guidelines.’ (P9, 32-year-old, Male)

‘We have soft copies on the phones and some consultations rooms have hard copies for references.’ (P7, 45-year-old, Female)

**The role of training on pre-exposure prophylaxis or antiretroviral treatment guidelines:** Participants highlighted that formal training on NIMART and PrEP guidelines played a crucial role in preparing them for effective PrEP implementation. They found that the integration of PrEP guidelines into the updated NIMART framework enhanced their readiness, equipping them with the necessary knowledge and skills to implement these guidelines confidently and efficiently:

‘Well, PrEP medication is part of ARV’s, so we use our knowledge from NIMART to implement PrEP, and with new ART guidelines and family planning trainings a little bit of PrEP is incorporated in those trainings. So, they provide minimal information about PrEP, they don’t go in details. They give us basic information about PrEP.’ (P1, 37-year-old, Female)

‘I cannot say I was prepared, but because I am NIMART trained, and I have experience in ART I just took over. At times you find that the patients come, and they need PrEP. The nurse who is trained is occupied with other patients or is not there so I felt that with my knowledge I can do this and that’s how I did it.’ (P19, 49-year-old, Female)

**Access to effective referral system and laboratory services:** Participants reported that a clear referral pathway enabled them to carry their duty effectively. They emphasised that having an established referral system at both the current level of care and the next level, along with access to laboratory facilities, greatly facilitated the referral of patients with complications to the appropriate level of care. The following are participants’ direct quotes:

‘However, most of the time when one meets the challenges with patients, we consult one another in the facility and for patients with complications we refer them to hospital for further management.’ (P3, 49-year-old, Female)

Other participants reported that they have quick access to the laboratory, which assists them to manage patients effectively because they come twice a day for the sample collection. Participants cited that having direct access to laboratory services such as E-LAB enable them to obtain blood results quick:

‘We don’t encounter many problems with Lab because they do come and collect specimen. They come in the morning and afternoon. And if they didn’t come, we store blood specimens in the fridge, and they come take them the next morning. The turnaround time is 48 hours, and we have access to blood results, and we also get the hardcopies. We can also get the results from the lab track system before we get the hardcopies.’ (P8, 31-year-old, Male)

#### Theme 3: Unclear performance tracking (outcome)

Participants reported unclear performance tracking as a significant barrier to PrEP programme, highlighting uncertainty regarding facility targets for PrEP uptake. Participants acknowledged the significance of measuring PrEP uptake as a vital indicator of programme success; however, they expressed uncertainty regarding their specific clinic targets. The absence of clarity resulted in uncertainty regarding the clinic’s success in achieving its targets, which may affect the evaluation and improvement of the programme’s implementation.

**Uncertainty regarding facility PrEP targets:** Participants indicated that they are not sure about the uptake or retention of the people on PrEP, thus do not know the outcome of their guideline implementation. Most of them stated that they were not sure what their weekly or monthly PrEP target was. However, they knew that they had targets to meet weekly and monthly. The following are the supporting statements:

‘I don’t want to lie to you, I don’t know how the clinic is performing with regards to PrEP target.’ (P1, 37-year-old, Female)

‘I don’t know it now but in 2020, when I was an acting operational manager, it was 12 patients a week.’ (P3, 49-year-old, Female)

## Discussion

This study sought to explore the availability of resources necessary for assisting nurses in the implementation of PrEP guidelines across PHC facilities in Johannesburg, utilising the Donabedian model as an analytical framework. The results identified several resource-related facilitators and barriers in the areas of structure, process and outcomes that play a role in the implementation of PrEP guidelines. The study highlights importance of resource availability in HIV prevention success.

### Structural inadequacies (structure)

The study revealed ongoing infrastructural limitations, such as inadequate consultation rooms and overcrowded clinic settings. The size of the facilities was small to handle the patient volume, thereby impacting workflow, privacy and patient satisfaction. The challenges observed align with earlier studies in South Africa that link inadequate physical infrastructure to ineffective service delivery in HIV prevention and treatment programmes (Malakoane et al. 2020; Rasesemola 2023). Comparably, a similar situation was noticed in a study conducted in Uganda, where the facility staff consistently reported having sufficient space and infrastructure to implement the intervention (Thomas et al. 2022).

Furthermore, successful implementation of PrEP guidelines is directly linked to the availability of resources such as staff members. This study found that staff shortage is a significant barrier to the success of this programme, impacting patient-to-clinician ratios and leading to increased workloads, heightened stress and diminished service quality. The findings are consistent with Liu, Zhang and Chen (2024), who identified burnout in overextended healthcare workers, which adversely affects care delivery. The shortage of human resources constrains outreach, education and patient retention efforts, all of which are critical for the effective implementation of PrEP. Human resources seem to be a common challenge in other African countries as well, impacting the implementation of PrEP. This is observed in studies conducted in Kenya, Uganda and other Western African countries (Admassu, Nöstlinger & Hensen et al. 2024; Thomas et al. 2022).

Clinical mentorship plays a significant role in improving the quality of healthcare service deliver (Mikkonen et al. 2022). The findings of this study revealed that the presence of clinical mentors played a crucial role to provide structural support, despite the challenges encountered. Mentors provided guidance on intricate cases and enhanced nurses’ ability to implement guidelines effectively. Despite infrequent physical visits, mentors offered significant telephonic support, highlighting the necessity of continuous and accessible mentorship systems in the implementation of PrEP programmes (Núñez et al. 2017; Nelson et al. 2023). Rwanda implemented a clinical mentorship programme that aimed to improve HIV service delivery, similar to what is done in South Africa, where clinical mentors play a crucial role in addressing barriers related to the implementation of PrEP, thus ensuring the quality of the programme (Sebeza et al. 2022; Visser et al. 2018). This result highlights the importance of ongoing mentorship to ensure PrEP implementation success.

### Healthcare system processes and support (process)

The study identified notable deficiencies in data management, especially concerning the TIER.Net system. Participants indicated a prevalence of inaccuracies, absent records and challenges in monitoring patient outcomes. The findings align with those of Huber et al. (2022) and Etoori et al. (2020), who similarly identified limitations of TIER.Net in accurately representing treatment data. The challenges observed are not exclusive to South Africa; studies conducted in Kenya and Uganda (Owaraganise et al. 2021) similarly indicate that electronic health systems face difficulties in real-time PrEP monitoring, attributed to incomplete data entries and insufficient interoperability among platforms. In contrast, high-income countries such as the United States utilise integrated electronic medical records that automatically identify missed appointments or gaps in lab monitoring (Spinelli et al. 2020), highlighting disparities in digital infrastructure that affect the fidelity of PrEP programmes. In this study, a disorganised file system hindered tracking, highlighting the necessity for more reliable health information systems and streamlined data processes to enhance continuity of care and monitoring.

Training emerged as a crucial facilitator for the implementation of PrEP. Nurses with formal training demonstrated increased confidence and competence in service delivery, aligning with the findings of Solomons et al. (2019), which indicate that HIV-specific training enhances provider knowledge and service quality. However, a significant gap existed as some participants depended on previous ART or NIMART training, which contained limited information on PrEP. This is similarly reflected in the findings of a study conducted in Lesotho (Matekane, Mohale & Khobotlo 2021), where healthcare workers reported discomfort in initiating PrEP because of deficiencies in training, despite the presence of robust national guidelines. Programmes in Thailand and Australia have indicated that regular training updates and supportive supervision have enhanced nurse-led PrEP success (Holt et al. 2021), emphasising the necessity of a focused training agenda for PrEP scale-up. This underscores the necessity for focused and thorough PrEP training for nurses, HIV counsellors and data personnel.

This study demonstrated an optimistic view of laboratory service integration, with access to the National Health Laboratory Service (NHLS) platforms such as Lab-Track facilitating result retrieval. Timing issues, including delays in sample collection and insufficient overnight storage, hindered effective monitoring. Comparable limitations were observed in Nigeria and Malawi (Nkoloma-Karimunda et al. 2020), where specimen logistics in rural clinics caused delays in results, frequently resulting in patient loss to follow-up. This differs from high-resource environments where point-of-care creatinine testing and same-day initiation minimise delays (Marcus et al. 2019). These disparities indicate that structural investments in diagnostic infrastructure are essential for enhancing patient retention and ensuring timely PrEP monitoring.

Referral pathways were essential in the context of this study, with peer consultations and intra-facility collaboration effectively facilitating routine care. However, complex patients required referrals to external hospitals, which sometimes delayed continuity of care. This illustrates findings from rural Mozambique (Geldsetzer et al. 2021), indicating that restricted access to specialists hindered timely management of urgent PrEP-related issues. In contrast, decentralised PrEP models in Brazil and Canada utilise telehealth and mobile consultations to enhance referral networks (Tan, Berchtold & O’Hara 2021), illustrating how innovations can address system-level barriers in resource-limited settings.

### Unclear performance tracking (outcome)

A significant observation was the lack of awareness among nurses concerning the PrEP initiation targets at their facilities. Participants acknowledged the presence of targets; however, many lacked awareness of the specific figures, indicating a broader issue in performance feedback and data transparency. This is consistent with the findings of Maseko, Mohlabane and Mokhele (2021) in South Africa, which indicated that nurses participating in HIV prevention programmes frequently lacked clarity regarding key performance indicators, thereby hindering their capacity for self-assessment and enhancement of service delivery.

The lack of target awareness may impede strategic planning, resource optimisation and individual accountability, thereby impacting patient uptake and retention. Odhiambo, Mwau and Ojoo (2020) found in Kenya that inadequate communication of performance metrics led to demotivation among health workers and resulted in inconsistent delivery of PrEP. Conversely, study conducted in the United States and Australia indicates that regular feedback techniques and data dashboards can markedly enhance staff engagement and programme outcomes (Grulich et al. 2021; Sharma et al. 2020). These systems enhance transparency and foster a culture of ongoing quality improvement.

The observed gap between facility-level targets and frontline staff awareness indicates insufficient internal communication and poor integration of monitoring and evaluation (M&E) frameworks. The misalignment poses significant challenges in decentralised health systems, where effective task-shifting to nurses necessitates robust supervision and precise goal setting for successful implementation. Kamwendo et al. (2022) highlighted that regular staff briefings regarding PrEP targets, progress and challenges promote a shared commitment to HIV prevention objectives in Malawi. This supports the notion that reinforcing internal communication channels is essential for empowering nurses to contribute meaningfully to national HIV prevention strategies.

### Strengths and limitations

This study’s primary strength is in its application of the Donabedian framework to systematically assess resource availability, facilitating straightforward interpretation across all dimensions of healthcare service. Furthermore, integrating the viewpoints of nurses, the primary implementors of PrEP services, enhances the practical understanding of the available evidence about PrEP implementation challenges. The incorporation of clinics from several sub-districts with heterogeneous patient volumes augments the applicability of the findings to wider urban healthcare settings in South Africa.

However, there are limitations too. Participants were drawn from only four clinics out of the 16 clinics in sub-district A and E; therefore, generalisability is limited. Additionally, the study focused on a specific context in Johannesburg; therefore, applicability to other contexts may not be possible.

## Conclusion and recommendations

This study identified significant structural, procedural and outcome-related challenges that hinder nurses’ effective implementation of PrEP guidelines. Mentorship, laboratory access and training are identified as facilitators to the implementation of PrEP guidelines. However, ongoing deficiencies in infrastructure, human resources, data systems and target communication constitute significant barriers. The findings support the study’s objective and indicate that successful implementation of PrEP at the facility level requires substantial investments in personnel, infrastructure, training and health system coordination. Enhancing these areas may substantially improve PrEP uptake and retention, thereby aiding South Africa in its objective of attaining an HIV-free generation by 2030.

Policymakers should focus on improving healthcare infrastructure and increasing the workforce to minimise delays and inefficiencies in the delivery of PrEP and ART services. Additionally, consistent training for healthcare providers on PrEP and ART guidelines is vital to enhance their ability to offer high-quality care. Strengthening data management systems is crucial for tracking PrEP uptake and retention, enabling evidence-based decision-making. Reliable data can guide the scaling up of programmes and help identify priority areas for intervention. Future research should explore the long-term cost-effectiveness of investing in healthcare systems and their impact on achieving HIV programme goals.
